# Impact of mammographic screening on ethnic and socioeconomic inequities in breast cancer stage at diagnosis and survival in New Zealand: a cohort study

**DOI:** 10.1186/s12889-015-1383-4

**Published:** 2015-01-31

**Authors:** Sanjeewa Seneviratne, Ian Campbell, Nina Scott, Rachel Shirley, Ross Lawrenson

**Affiliations:** Waikato Clinical School, University of Auckland, Auckland, New Zealand; Department of Surgery, University of Colombo, Colombo, Sri Lanka; Māori Health Services, Waikato District Health Board, Hamilton, New Zealand; Waikato Breast Cancer Trust, Waikato Hospital, Hamilton, New Zealand; Breast Cancer Research Office, Waikato Hospital, PO Box 934, Hamilton, 3240 New Zealand

**Keywords:** Breast cancer, Screening, Ethnicity, Deprivation, Inequity

## Abstract

**Background:**

Indigenous Māori women experience a 60% higher breast cancer mortality rate compared with European women in New Zealand. We explored the impact of differences in rates of screen detected breast cancer on inequities in cancer stage at diagnosis and survival between Māori and NZ European women.

**Methods:**

All primary breast cancers diagnosed in screening age women (as defined by the New Zealand National Breast Cancer Screening Programme) during 1999–2012 in the Waikato area (n = 1846) were identified from the Waikato Breast Cancer Register and the National Screening Database. Stage at diagnosis and survival were compared for screen detected (n = 1106) and non-screen detected (n = 740) breast cancer by ethnicity and socioeconomic status.

**Results:**

Indigenous Māori women were significantly more likely to be diagnosed with more advanced cancer compared with NZ European women (OR = 1.51), and approximately a half of this difference was explained by lower rate of screen detected cancer for Māori women. For non-screen detected cancer, Māori had significantly lower 10-year breast cancer survival compared with NZ European (46.5% vs. 73.2%) as did most deprived compared with most affluent socioeconomic quintiles (64.8% vs. 81.1%). No significant survival differences were observed for screen detected cancer by ethnicity or socioeconomic deprivation.

**Conclusions:**

The lower rate of screen detected breast cancer appears to be a key contributor towards the higher rate of advanced cancer at diagnosis and lower breast cancer survival for Māori compared with NZ European women. Among women with screen-detected breast cancer, Māori women do just as well as NZ European women, demonstrating the success of breast screening for Māori women who are able to access screening. Increasing breast cancer screening rates has the potential to improve survival for Māori women and reduce breast cancer survival inequity between Māori and NZ European women.

## Background

Breast cancer is the most common cancer in New Zealand women, a rate which is the ninth highest in the world [1]. The burden of breast cancer has an unequal ethnic distribution in New Zealand as Indigenous Māori women have a higher incidence and a lower survival rate compared with NZ European women [2]. Many factors are believed to be contributory to worse breast cancer outcomes for Māori compared with NZ European women including delay in diagnosis, inferior quality and delays in treatment, and higher rates of comorbidity.

In parallel with many other developed countries, New Zealand has experienced a substantial reduction in breast cancer mortality over the last two decades [1]. While some of the observed reduction in breast cancer mortality in these countries has been attributed to advances in treatment, mammographic breast cancer screening has also played a major role [3]. BreastScreen Aotearoa (BSA) is the New Zealand National Breast Screening Programme, a free mammographic breast screening service available for all ‘screening age’ women. Since it was established in 1999, the BSA has provided free biennial mammographic screening for all women aged between 50 to 64 years and this age range was extended to include women aged 45 to 49 and 65 to 69 years in July 2004.

Mammographic screening coverage in New Zealand has gradually picked up over the last decade and has achieved the target biennial coverage of 70% for NZ European women since 2010 [4]. However, poor screening coverage has remained a significant issue for Māori women for whom the coverage was only 62.7% in 2012 [4], well below the 70% target coverage. Further, there was a large variability in screening coverage rate for Māori by region, which ranged from 54% to 79% across the country in 2012 [5]. There is, however, close monitoring of Māori coverage, and targets are set yearly with screening providers to improve this. It is this effort that has picked Māori coverage up from below 40% at the commencement of BSA programme in 1999 to the current level of over 60% [5]. In spite of this, lower screening coverage remains a likely major contributor to inequities in breast cancer burden between Māori and NZ European women, as Māori have a higher breast cancer incidence and are more likely to present with advanced cancer compared with NZ European women [1]. We conducted this study to explore differences in rates of screen detected cancer by ethnicity and socioeconomic deprivation in a cohort of screening age women, and to determine the contribution of these differences to ethnic and socioeconomic inequities in breast cancer survival in New Zealand.

## Methods

### Study population

Data for this study were obtained from the Waikato Breast Cancer Registry (WBCR). The WBCR is a prospectively maintained population based regional cancer registry that includes in-situ and invasive breast cancers diagnosed in the Waikato District Health Board area since 1999. The WBCR includes over 98% of all diagnosed cancers in the region (public and private) and validity of its data has been reported previously [6]. The Waikato District Health Board covers an area with a population of approximately 380,000 of whom 21% are Māori. This is the second largest regional Māori population in New Zealand [7].

All screening age women with newly diagnosed breast cancer between 01/01/1999 and 31/12/2012 were eligible for this study (n = 1846). Screening age was defined according to the BSA. This included women between 50 and 64 years up to June 2004 and women between 45 and 69 years from July 2005 onwards. Screening status was classified into screen-detected (n = 1064, 57.6%), interval (if diagnosis within 24 months from last screening mammogram, n = 241, 13.1%) and non-interval symptomatic (n = 541, 29.3%). Cancers diagnosed through BSA (n = 954, 89.7%) and through opportunistic screening mammograms arranged by physicians outside BSA (n = 110, 10.3%) were included under screen detected cancers. Screening status for each woman diagnosed through BSA was confirmed by comparing with screening data from the BSA database, which include data (i.e., screen detected and interval) for all women with breast cancer diagnosed through the BSA programme. Details of opportunistic screening were based on the WBCR records, and were reconfirmed by accessing clinical and mammographic records of all these women. For women with more than one episode of breast cancer during the study period, only the first cancer was included for analysis.

### Study covariates

Ethnicity of each woman was obtained from the WBCR which records self-identified ethnicity collected as a part of the WBCR consent process. Ethnicity was categorized in to NZ European, Māori, Pacific and Other for analysis. Deprivation was measured using the New Zealand Deprivation Index 2006 (NZDep2006), an area-based measure of socioeconomic status [8]. The NZDep2006 assigns small areas of residence, a deprivation decile on a scale of 1 to 10 based on nine socio-economic variables measured during the 2006 population census. Deciles were combined to create deprivation quintiles; 1–2 (least deprived), 3–4, 5–6, 7–8, and 9–10 (most deprived). Residential status was classified as urban, semi-urban or rural based on New Zealand Statistics urban rural classification system [9]. Cancer stage at diagnosis was defined according to the Tumour, Node, and Metastasis (TNM) staging system [10].

### Outcome variables

Date and cause of death for all deceased women (censored at 31/12/2013) were identified from the WBCR and from the Mortality Collection of the Ministry of Health. Follow up duration was calculated from the date of diagnosis to the date of death, or to the date of the last follow up when the patient was known to be alive (censored at 31/12/2013).

### Statistical analysis

Chi squared (*χ*^2^) test for trend was used to test for univariate differences in early versus advanced stage at diagnosis. Multivariate logistic regression analyses were used to test for differences in early versus advanced stage at diagnosis between Māori and NZ European women sequentially adjusting for age, year of diagnosis, screening status, socioeconomic deprivation and residential status. Impact of differences in screen detected cancer towards mortality disparity between Māori and NZ European women was explored in Cox proportional hazard models sequentially adjusting for same covariates. Interaction terms were included into regression models to identify possible interactions between ethnicity, deprivation and screening status (i.e., ethnicity*deprivation, ethnicity*screening and deprivation*screening). We also investigated 5-year and 10-year breast cancer specific survival rates for invasive cancers by screening status, ethnicity and socioeconomic deprivation using Kaplan-Meier survival curves. Survival comparisons by ethnicity were performed for Māori and NZ European women. Pacific and Other ethnic group women were excluded from these analyses. Statistical analyses were performed in SPSS (Version 22).

## Results

This study included a total of 1846 screening age women (1548 invasive and 298 in-situ) with newly diagnosed first primary breast cancer over the study period. Of these women 1064 (57.6%) were screen detected and 782 (42.4%) were symptomatic. Of symptomatic women, 241 (30.8%) had interval and 541 (69.2%) had non-interval symptomatic cancers. Mean follow-up was 65.5 months (median 58 months). Sixty-seven percent of women were followed up for a minimum of five years or until death and 32% were followed up for ten years or until death. Overall mean age of included women was 56.8 years (median 56). Mean ages for in-situ and invasive breast cancers were 56.5 (median 56.5) and 56.9 years (median 56) respectively.

Lower proportions of cancers were observed within 45–49 and 65–69 age categories as these two age categories were included in the BSA only from July 2004 onwards (Table 1). When women diagnosed since July 2004 were considered alone, almost similar proportions of women were observed to be included in each age category, which ranged from 19.0% (55–59 age category) to 21.1% (60–64 age category).

**Table 1:** **Factors associated with early versus advanced stage at diagnosis of breast cancer by ethnicity**

		**NZ European**		**Māori**		**All cancers**				
**Characteristic**		**(N = 1459)**	**Early** ^**a**^	**(N = 311)**	**Early** ^**a**^	**(N = 1846)**	**Early** ^**a**^			
		**n (%)**	**n (%)**	**n (%)**	**n (%)**	**n (%)**	**n (%)**	**OR**	**95% CI**	**p**
Screening status										<0.001
	Screen detected	915 (62.7)	654 (71.5)	153 (49.2)	107 (69.9)	1106 (59.9)	786 (71.1)	Ref		
	Interval	192 (13.2)	63 (32.8)	20 (6.4)	3 (15.0)	218 (11.3)	69 (31.7)	5.30	4.15-6.74	
	Non-screen	352 (24.1)	118 (33.5)	138 (44.4)	36 (26.1)	522 (28.3)	162 (31.0)	5.46	4.11-7.33	
Age group (years)										
	45-49	208 (14.3)	110 (52.9)	51 (16.4)	23 (45.1)	275 (14.9) ^b^	143 (52.0)	Ref		0.005
	50-54	375 (25.7)	206 (54.9)	77 (24.8)	31 (40.3)	469 (25.4)	245 (52.2)	0.99	0.74-1.33	
	55-59	323 (22.1)	180 (55.7)	74 (23.8)	32 (43.2)	416 (22.5)	218 (52.4)	0.98	0.73-1.33	
	60-64	337 (23.1)	196 (58.2)	73 (23.5)	36 (49.3)	425 (23.0)	241 (56.7)	0.83	0.61-1.12	
	65-69	216 (14.8)	143 (66.2)	36 (11.6)	24 (66.7)	261 (14.1) ^b^	170 (65.1)	0.58	0.41-0.82	
Deprivation										
	Dep 1-2	184 (12.6)	101 (54.9)	9 (2.9)	1 (11.1)	203 (11.0)	108 (53.2)	Ref		0.094
	Dep 3-4	157 (10.8)	104 (66.2)	23 (7.4)	13 (56.5)	186 (10.1)	120 (64.5)	0.63	0.42–0.94	
	Dep 5-6	339 (23.2)	189 (55.8)	62 (19.9)	29 (46.8)	411 (22.3)	223 (54.3)	0.96	0.68–1.34	
	Dep 7-8	492 (33.7)	279 (56.7)	95 (30.5)	43 (45.3)	614 (33.3)	338 (55.0)	0.93	0.68–1.28	
	Dep 9-10	287 (19.7)	162 (56.4)	122 (39.2)	60 (49.2)	432 (23.4)	228 (52.8)	1.02	0.73–1.42	
Residence										
	Urban	783 (53.7)	435 (55.6)	150 (48.2)	75 (50.0)	990 (53.6)	539 (54.4)	Ref		0.794
	Semi-urban	370 (25.4)	222 (60.0)	107 (34.4)	50 (46.7)	492 (26.7)	277 (56.3)	0.93	0.75–1.15	
	Rural	306 (21.0)	178 (58.2)	54 (17.4)	21 (38.9)	364 (19.7)	201 (55.2)	0.97	0.76–1.23	
Year of diagnosis										
	1999-2002	249 (17.1)	128 (51.4)	44 (14.1)	20 (45.5)	300 (16.3)	153 (51.0)	Ref		0.423
	2003-2006	450 (30.8)	270 (60.0)	75 (24.1)	33 (44.0)	542 (29.4)	308 (56.8)	0.79	0.60-1.05	
	2007-2009	363 (24.9)	203 (55.9)	86 (27.7)	45 (52.3)	479 (25.9)	263 (54.9)	0.86	0.64-1.14	
	2010-2012	397 (27.2)	234 (58.9)	106 (34.1)	48 (45.3)	525 (28.4)	293 (55.8)	0.82	0.62-1.09	

(Characteristics of women associated with early stage [compared with advanced stage] at diagnosis of breast cancer by ethnicity for screening age women with newly diagnosed breast cancer in the Waikato, New Zealand 1999–2012).

(^a^early stage = in-situ & stage I, ^b^only cancers diagnosed from July 2004 onwards are included).

No difference in age distribution was seen for Māori and NZ European women where the median age at diagnosis was 56 years. Women of higher socioeconomic deprivation quintiles were significantly older at diagnosis compared with women of lower deprivation quintiles which ranged from a mean age of 55.2 years for deprivation quintile 1–2 to a mean age of 57.2 years for deprivation quintile 9–10 (p < 0.001).

Overall, 62.7% of all breast cancers among NZ European women within screening age were screen detected compared with 49.2% among Māori women of this age range (p < 0.001). Of the screen detected cancers, a higher proportion of cancers in NZ European women were detected through opportunistic screening (10.9%) compared with Māori (7.3%), but this difference was statistically not significant (p = 0.183). The difference in proportion of screen detected cancer between NZ European and Māori women was significant for invasive cancers (58.3% vs. 45.1%, p = <0.001), but was not statistically significant for in-situ cancers (85.4% vs. 74.4%, p = 0.063).

Univariate analysis of factors associated with early stage disease (in-situ & stage I, n = 1017) at diagnosis compared with more advanced stage (stages II, III & IV, n = 829) is shown in Table 1. Non-screen compared with screen detection was significantly associated with more advanced stage at diagnosis (OR = 5.46, p < 0.001) as did Māori compared with NZ European ethnicity (OR = 1.51, 95% CI 1.18-1.93, p = 0.001). No significant differences were observed in early versus advanced stage at diagnosis by deprivation status (p = 0.094). Table 2 shows odds ratios from multivariate logistic regression analyses for advanced versus early stage at diagnosis in Māori compared with NZ European women with sequential adjustment for covariates. Adjusting for screening status reduced the odds ratio for more advanced stage at diagnosis in Māori compared with NZ European from 1.49 (1.15-1.91) to 1.25 (0.96-1.64). A minimal further attenuation in odds ratio was observed with additional adjustments for socioeconomic deprivation and residential status (OR = 1.24, 0.95-1.65). Similarly, odds of advanced stage at diagnosis remained largely unchanged after introducing interaction terms into the model (OR = 1.28, 0.74-2.23). In a separate analysis (data not shown), socioeconomic deprivation was introduced into the model prior to screening status, but made no difference to the odds ratio (OR = 1.49, 1.16-1.93).

**Table 2:** **Factors associated with advanced stage at diagnosis of breast cancer in Māori compared with NZ European women**

**Characteristic**	**OR**	**95% CI**	**p**
Model A (Unadjusted))	1.51	1.18–1.93	0.001
Model B (Age adjusted)	1.49	1.16-1.91	0.002
Model C (Model B + Year of diagnosis ^c^)	1.49	1.15-1.91	0.002
Model D (Model C + Screening status)	1.25	0.96-1.64	0.101
Model E (Model D + Deprivation)	1.24	0.94-1.64	0.133
Model F (Model E + Urban/Rural residence)	1.24	0.95-1.65	0.125
Model H (Model E + interaction terms ^d^)	1.28	0.74-2.23	0.373

(Odds ratios for stage at diagnosis (i.e., advanced ^b^versus early ^a^) in Māori compared with NZ European women with stepwise adjustment for age, year of diagnosis, screening status, socioeconomic deprivation and urban/rural residential status).

(^a^early stage = in-situ & stage I, ^b^advanced stage = stages I to III, ^c^year categories as in Table 1, d – ethnicity x deprivation, ethnicity x screening and deprivation x screening).

Next we repeated a similar regression analyses only including invasive cancers to identify factors associated with more advanced invasive cancer (stages II, III & IV, n = 829) versus early invasive (stage I, n = 719) cancer at diagnosis (data not shown). This analysis yielded results similar to initial analysis with Māori having significantly more advanced disease at diagnosis (unadjusted) compared with NZ European women (OR = 1.53, 1.17-2.01, p = 0.002). Adjusting for screening status resulted in a reduction of age and year adjusted odds ratio for advanced cancer in Māori compared with NZ European from 1.52 (1.15-1.99) to 1.30 (0.97-1.75). Further adjustment for deprivation made no difference to this odds ratio (OR = 1.30, 0.96-1.75).

Table 3 shows adjusted breast cancer specific mortality hazard ratios from Cox regression models by screening status for women with invasive breast cancers. Maori women were observed to have higher hazards of breast cancer mortality overall (HR = 1.33, 0.33-3.18) and for screen detected (HR = 1.45, 0.33-6.39) and non-screen detected cancers (HR = 3.13, 1.58-6.18), although this was statistically significant only for non-screen detected cancer. Breast cancer specific mortality hazard ratios for Māori compared with NZ European women with sequential adjustment for covariates is shown in Table 4. In the model for all cancers, adjusting for screening status reduced the mortality hazard for Māori from 2.33 to 2.01, while further adjusting for deprivation resulted in a marginal increase of hazard up to 2.09. Adjusting for socioeconomic deprivation before adjusting for screening (data not shown) saw a similar increase in hazard for Māori from 2.33 to 2.37. For screen detected cancers, Māori women had non-significant lower hazards of mortality before (HR = 0.77) and after adjusting for covariates (HR = 0.85), but was non-significantly higher after introduction of interaction terms (HR = 1.45). In contrast, for non-screen detected cancers Māori had a significantly higher risk of mortality which was more than double that for NZ European women before (HR = 2.28) and after adjusting for covariates (HR = 2.37) and introduction of interaction terms (HR = 3.13).

**Table 3:** **Breast cancer specific mortality hazard ratios from Cox regression model (Adjusted for age and year of diagnosis)**

		**All cancers**			**Screen detected**			**Non-screen detected**		
**Characteristic**		**HR**	**95% CI**	**p**	**HR**	**95% CI**		**HR**	**95% CI**	**p**
Ethnicity										
	NZ European	Ref			Ref			Ref		
	Māori	1.33	0.33-3.18	0.964	1.45	0.33-6.39	0.620	3.13	1.58-6.18	0.001
Mode of diagnosis										
	Screen detected	Ref			-			-		
	Non-Screen	2.81	1.57-5.04	0.001						
Deprivation quintile										
	1-2	Ref		0.118	Ref		0.627	Ref		0.019
	3-4	0.84	0.39-1.78		0.77	0.19-3.10		0.76	0.35-2.13	
	5-6	0.86	0.45-1.66		0.91	0.28-2.79		0.68	0.39-1.84	
	7-8	1.26	0.58-2.74		1.13	0.35-3.60		0.08	0.93-3.76	
	9-10	0.73	0.32-1.69		0.56	0.15-2.06		0.71	0.52-2.56	
Residential status										
	Urban	Ref		0.311	Ref		0.370	Ref		0.155
	Semi-urban	0.77	0.53-1.12		1.51	0.73-3.14		0.65	0.42-1.01	
	Rural	0.81	0.54-1.21		0.78	0.31-1.94		0.85	0.54-1.32	
Ethnicity x Deprivation		0.65	0.31-1.36	0.253	0.39	0.05-3.07	0.370	0.69	0.31-1.54	0.468
Ethnicity x Screening		3.29	1.10-9.85	0.033	-			-		
Deprivation x Screening		1.47	0.71-3.06	0.302	-			-		

(x – Interaction terms).

**Table 4:** **Hazard ratios for breast cancer-specific mortality risk in Māori compared with NZ European women**

**Characteristic**	**All cancers**		**Screen detected**		**Non-screen detected**	
	**HR (95% CI)**	**p**	**HR (95% CI)**	**p**	**HR (95% CI)**	**p**
Model A (Unadjusted))	2.25 (1.62-3.12)	<0.001	0.77 (0.27-2.15)	0.617	2.28 (1.59-3.26)	<0.001
Model B (Age adjusted)	2.29 (1.69-3.18)	<0.001	0.80 (0.29-2.25)	0.674	2.34 (1.63-3.35)	<0.001
Model C (Model B + Year of diagnosis^a^)	2.33 (1.64-3.25)	<0.001	0.84 (0.31-2.36)	0.738	2.27 (1.59-3.25)	<0.001
Model D (Model C + Screening status)	2.01 (1.44-2.80)	<0.001	-	-	-	-
Model E (Model D + Deprivation)	2.09 (1.49-2.94)	<0.001	0.85 (0.30-2.40)	0.762	2.39 (1.65-3.46)	<0.001
Model F (Model E + Urban/Rural residence)	2.11 (1.50-2.97)	<0.001	0.85 (0.30-2.41)	0.760	2.37 (1.64-3.47)	<0.001
Model G (Model F + Interaction terms^b^)	1.33 (0.33-3.18)	0.964	1.45 (0.33-6.39)	0.620	3.13 (1.58-6.18)	0.001

(With stepwise adjustment for age, year of diagnosis, screening status, socioeconomic deprivation and urban/rural residential status).

(^a^year categories as in Table 1, ^b^ – ethnicity x deprivation, ethnicity x screening and deprivation x screening.

Unadjusted five and 10-year breast cancer specific survival rates for invasive cancers by screening status, ethnicity and deprivation are shown in Table 5. Screen detected invasive cancers demonstrated the highest five and 10-year breast cancer specific survival rates and the lowest rates were seen for non-interval symptomatic breast cancers. Māori women had significantly lower crude five (90.2% vs. 77.6%) and 10-year (83.5% vs. 73.8%) breast cancer survival rates compared with NZ European women. Compared with women from more affluent (1–2, 3–4 to 5–6) quintiles, women from more deprived quintiles (7–8 and 9–10) had significantly worse 10-year breast cancer survival rates (84.3% vs. 77.2%, p = 0.011).

**Table 5:** **Breast cancer specific survival rates by screening status, ethnicity and socioeconomic deprivation**

**Characteristic**	**No. of women**	**Total breast cancer deaths**	**5-year survival**	**95% CI**	**10-year survival**	**95% CI**
Screening status						
Screen detected	858	40	96.2%	94.6 - 97.8	91.8%	88.3 - 95.3
Interval	217	36	83.9%	79.3 - 88.5	73.5%	64.1 - 82.9
Non-screen non-interval	473	108	76.4%	70.9 - 81.9	66.3%	60.2 - 72.4
Ethnicity						
NZ European	1220	126	90.2%	88.2 - 92.2	83.5%	77.4 - 89.6
Māori	268	50	77.6%	71.5 - 83.7	67.8%	58.4 - 77.2
Deprivation						
Dep 1-2	165	14	89.5	83.8 - 95.2	87.1	80.2 - 94.0
Dep 3-4	155	15	89.7	84.2 - 95.2	85.8	79.7 - 93.9
Dep 5-6	347	33	90.8	87.1 - 94.5	85.0	76.6 - 89.6
Dep 7-8	513	81	84.1	80.4 - 87.8	75.4	69.9 - 80.9
Dep 9-10	368	41	88.2	84.7 - 91.7	79.7	72.6 - 86.8

(Five-year and 10-year breast cancer specific survival rates by for screening age women with invasive breast cancer in the Waikato, New Zealand 1999–2012).

**Figure 1:**
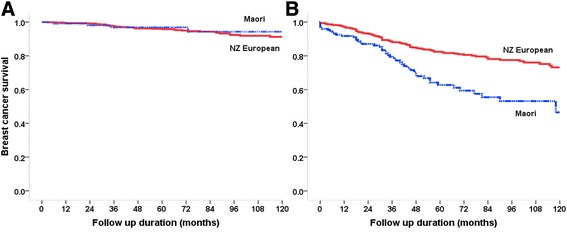
**Breast cancer specific survival by ethnicity and screening status.** (Kaplan-Meier survival curves for screen detected **(Panel A)** and non-screen detected **(Panel B)** breast cancers in screening age women by ethnicity in the Waikato, New Zealand 1999–2012).

Māori women with non-screen detected invasive cancer had significantly worse five and 10-year breast cancer survival rates compared with NZ European women (83.1% vs. 64.2% and 73.2% vs. 46.5% respectively, p < 0.001). In contrast, for screen detected breast cancer, Māori women had, if anything, better five and 10-year breast cancer specific survival rates (96.9% and 94.1%) compared with NZ European women (95.8% and 90.3%), although this was statistically non-significant (p = 0.651) (Figure 1).

**Figure 2:**
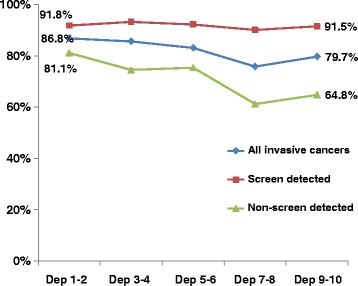
**Ten-year breast cancer specific survival rates by socioeconomic deprivation.** (Based on Kaplan-Meier survival curves by socioeconomic deprivation quintile for screening age women in the Waikato, New Zealand 1999–2012).

Figure 2 shows the association between screening status and socioeconomic deprivation on 10-year breast cancer specific survival rates based on Kaplan-Meier survival analysis. There was a tendency for higher survival in women from more affluent quintiles. The difference in breast cancer specific survival rates between more and less affluent women was substantially lower for women with screen detected breast cancer than for non-screen detected breast cancer. This has resulted in a greater survival difference between screen and non-screen detected cancer with increasing socioeconomic deprivation.

**Figure 3:**
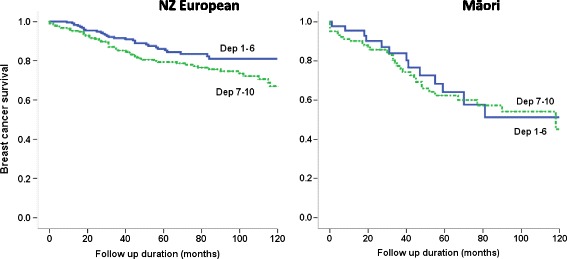
**Breast cancer specific survival by deprivation and ethnicity.** (Kaplan-Meier survival curves for non-screen detected cancers in screening age NZ European and Māori women by socioeconomic deprivation status in the Waikato, New Zealand 1999–2012).

Figure 3 illustrates breast cancer specific survivals for women with non-screen detected cancer by socioeconomic deprivation (Dep 1–6 versus Dep 7–10) and ethnicity. Among NZ Europeans, women of higher socioeconomic groups (Dep 1–6) had significantly better survival rates compared with women of lower socioeconomic groups (Dep 7–10) (10-year survival 81% vs. 67.6%, p = 0.026). In comparison, survival rates did not vary by deprivation status among Māori women with non-screen detected cancer (10-year survival 51.2% vs. 44.4%, p = 0.715).

## Discussion

From this study we found that breast cancer survival inequities between Māori and NZ European women in the screening age group were significant for women diagnosed through the non-screen pathway but absent for women diagnosed through screening. Five and 10-year survival for women diagnosed through screening were 2.8% and 5.2% higher respectively for Māori compared with NZ European women, while Māori women diagnosed through the non-screen pathway had a 9.9% lower 5-year and a 17.7% lower 10-year survival than NZ European women. We found that patterns of inequities by socioeconomic deprivation were similar to those found between Māori and NZ European women. For non-screen detected cancer, 10-year breast cancer specific survival was 16.3% lower for most deprived compared with women from most affluent socioeconomic quintile.

Benefits of population based mammographic breast cancer screening have been proven by several randomized trials [3,11]. It has been shown that provision of biennial screening with a 70% coverage confers an approximately 30% reduction in breast cancer mortality for the screening population [12]. The breast cancer incidence for NZ European women is similar to the rates seen in breast cancer screening trials [12,13], but incidence in Māori is much higher [14]. Hence, at a 70% biennial screening rate, more Māori women may benefit than non-Māori, and given that Māori women with symptomatic cancer tend to present at a later stage than non-Māori, Māori women may also have a greater reduction in breast cancer mortality with screening. Our data lend support this hypothesis.

Compared with NZ Europeans, a higher proportion of breast cancer is observed among younger Māori women due to the younger age structure of Māori population [15]. Further, the screen detection rate for Māori women between 45 to 49 years in initial mammographic screens is more than twice that for non-Māori, and is 50% greater even for subsequent screening mammograms [5]. Breast cancer in younger women tends to be more aggressive and is more likely to be associated with poorer outcomes [16]. Thus, it may be worthwhile exploring the usefulness of providing breast cancer screening for Māori women below the current screening age limit, for instance for women between 40 and 44 years. However, a thorough evaluation of potential benefits versus harms of mammographic screening should be an essential part of such an initiative.

Obese women are more likely to present with more advanced breast cancer compared with non-obese women and are more likely to be diagnosed through screening mammography than non-obese [17]. Approximately 48% of adult Māori women are obese, which is almost twice the rate observed in NZ European women [18]. Evidence from the USA suggests that up to 30% of later stage breast cancer diagnosed in African American women compared with White American women are attributable to higher rates of obesity in African American women [19]. At present, no New Zealand data are available on the impact of obesity on ethnic differences in breast cancer stage at diagnosis or outcomes. Nonetheless, higher obesity prevalence is likely to be another factor that would confer a greater benefit from increased mammographic screening coverage for Māori compared with NZ European women.

Survival differences for screen detected breast cancer were not seen, either by ethnicity or socioeconomic deprivation, while differences in survival were marked and significant for non-screen detected cancer by both. Similar observations have been reported from the UK, the USA and the Netherlands [14,20,21]. Inequities in breast cancer survival by ethnicity and socioeconomic status for women diagnosed through non-screen pathway are likely due to a range of factors. While later stage at diagnosis for Māori and women of socioeconomically deprived groups is the likely major factor for these differences, higher rates of obesity, comorbidities and differences in treatment compared with NZ European and more affluent women, respectively are also likely to be important. In addition, women who participate in mammographic screening may be more likely to have better access to health care, education, health literacy and health seeking behaviours compared with women from similar ethnic or socioeconomic backgrounds, who do not participate in screening [22].

Furthermore, there is a stringent quality framework and an audit process for the treatment of breast cancer detected through BSA, but not for cancers diagnosed outside BSA [4]. For instance, all women with BSA detected breast cancers are reviewed through a multidisciplinary meeting, and according to BSA quality standards, at least 90% of BSA detected women are expected to receive their first surgical treatment within 20 working days of receiving their final diagnostic result. Results for each regional screening provider are regularly audited and a feedback process ensures that adequate measures are undertaken by providers who fail to achieve these targets. Similar audit processes or quality frameworks are not in place for non-screen detected cancer treatment. Lack of a framework is a likely key reason for the disparities observed amongst women with non-screen detected cancers by ethnic and socioeconomic grouping. This is further supported by previous work which was based on the Waikato Breast Cancer Register on delay for primary surgical treatment of breast cancer [23]. This study showed significantly longer delays for Māori compared to NZ European women for non-screen detected breast cancer, but not for cancers diagnosed through BSA. To overcome these disparities in cancer care, the Ministry of Health is in the process of introducing quality measures for the management of all common cancers through the National Cancer Control Strategy. These include the Faster Cancer Treatment Indicators [24] and the Standards of Service Provision for Breast Cancer Patients in New Zealand [25]. These measures will provide benchmarks for quality cancer care and are expected to improve care for all women with breast cancer as well as reducing and hopefully eliminating inequities in access, timeliness and quality of care along the symptomatic breast cancer care pathway.

The main strengths of this study include the completeness and comprehensive nature of the study sample which include more than 98% of all breast cancers in eligible women over the study period. Data linkage with the national screening database enabled us to clearly define and validate screen detected, interval and symptomatic non-interval cancers. Furthermore, relatively longer follow up has provided fairly robust survival data with five and 10-year survival rates for groups of women of interest. Limitations of this study include the absence of data on previous breast cancer screening behaviours of these women, which is a factor known to be associated with cancer stage at diagnosis and long term outcomes [21]. Also, we were unable to ascertain the reasons for non-screening participation in women with symptomatic non-interval cancer, which however is beyond the scope of the present study. This study has used NZDep2006 as a measure of socioeconomic status, which is based on area level deprivation [8]. Although area level deprivation is not as accurate as individually measured socioeconomic status, NZDep2006 has been validated as an accurate proxy measure for assessment of individual deprivation [26].

## Conclusions

We have observed a significantly higher rate of advanced stage breast cancers among screening age Māori compared with NZ European women, of which approximately a half was explained by lower rate of screen detected cancer among Māori women. Significant differences in breast cancer survival by ethnicity and socioeconomic deprivation were observed for non-screen detected, but not for screen detected breast cancer. Māori women who do have screen detected breast cancers appear to do just as well as NZ European women demonstrating the success of BSA for Māori women who are able to access this programme. Achieving at least the national 70% biennial breast cancer screening rate for eligible Māori women will likely make a significant contribution to reducing cancer deaths for Māori as well as reducing inequities in cancer deaths between Māori and NZ European women in New Zealand.
